# Successful containment to date of SARS‐CoV‐2 transmission in the Northern Territory

**DOI:** 10.5694/mja2.50840

**Published:** 2020-10-28

**Authors:** Nicholas M Douglas, Ella M Meumann, Vicki L Krause, Jane Davies, for the Northern Territory COVID‐19 Response Group

**Affiliations:** ^1^ Royal Darwin Hospital Darwin NT; ^2^ Menzies School of Health Research Charles Darwin University Darwin NT; ^3^ Northern Territory Centre for Disease Control, Department of Health Darwin NT

**Keywords:** COVID‐19, Epidemiology, Signs and symptoms, Diagnosis, Public health, Virus diseases, Molecular medicine, Infectious diseases, Respiratory tract infections

Hospitals in the Northern Territory often operate beyond capacity and serve a sparsely distributed population with rates of chronic disease and household overcrowding that are higher than in many other parts of Australia. The NT consequently adopted particularly strict public health measures to avert the potentially catastrophic consequences of community transmission of severe acute respiratory syndrome coronavirus 2 (SARS‐CoV‐2), including supervised isolation until viral clearance of all people with confirmed SARS‐CoV‐2 infections (Supporting Information 1). This measure provided a unique opportunity to study the duration and trajectory of viral shedding in relation to clinical illness. In this article, we describe epidemiologic, clinical, and virological aspects of the first 28 cases of coronavirus disease 2019 (COVID‐19) in the NT. The Top End and Central Australian Human Research Ethics Committees approved the study (reference, 2020‐3737).

Between 4 March and 4 April 2020, 28 cases of COVID‐19 were diagnosed in the NT, all linked to overseas or interstate travel. The median age of patients was 45.0 years (range, 1.5–75 years); 16 were women (Supporting Information 1, table). Two patients required supplemental oxygen, one of whom also required intubation. There were no deaths.

Symptoms had been present for a median 3 days (range, 0–16 days) before oro‐nasopharyngeal swab collection and lasted a median 9.5 days (range, 4–18 days). Viral RNA could be detected by multiplex tandem real‐time polymerase chain reaction (PCR) assay (AusDiagnostics; Supporting Information 1) for a median 25 days after symptom onset (range, 14–41 days; interquartile range [IQR], 21–32 days), and in most patients for more than two weeks after symptom resolution (median, 17.5 days; range, 2–31 days; IQR, 14.5–22.5 days) (Box 1). Within‐patient variability in viral target cycle threshold values during follow‐up was considerable (Box 2; Supporting Information 1, figure), despite adequate and consistent amounts of human biologic material in test samples (data not shown). Prolonged compulsory isolation was distressing for several patients.

Box 1 **Time course of 28 cases of coronavirus disease 2019 (COVID‐19) diagnosed in the Northern Territory, 4 March – 4 April 2020**

Each line represents a single patient. Day zero is the day of collection of the first SARS‐CoV‐2‐positive specimen; thickened sections indicate the period of COVID‐19 symptoms. Closed circles indicate positive SARS‐CoV‐2 assay results, hollow circles negative assay results. Patients 13 and 15 (lighter marking) required supplemental oxygen. The bottom line summarises the median duration of symptoms prior to diagnosis, the median duration of symptoms, and the median time to viral clearance.

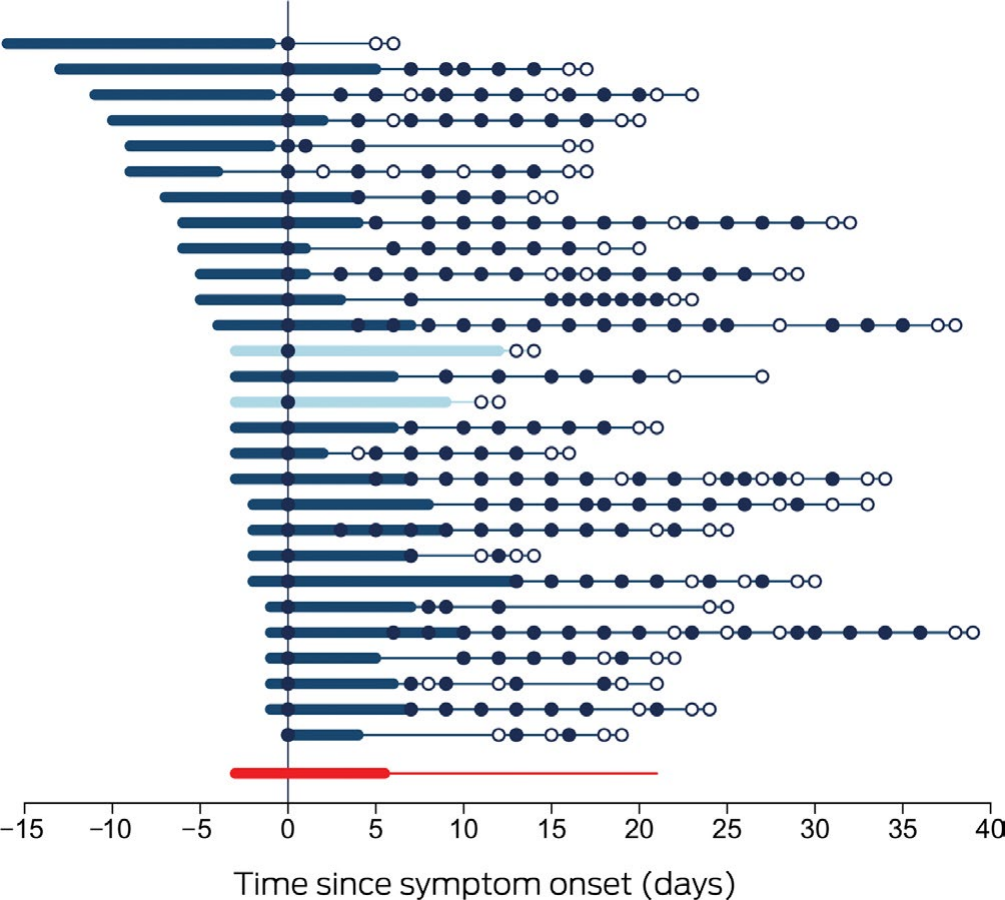

Box 2 **Multiplex tandem polymerase chain reaction cycle threshold values for detection of the SARS‐CoV‐2 open reading frame 1a gene (*ORF1a*)**

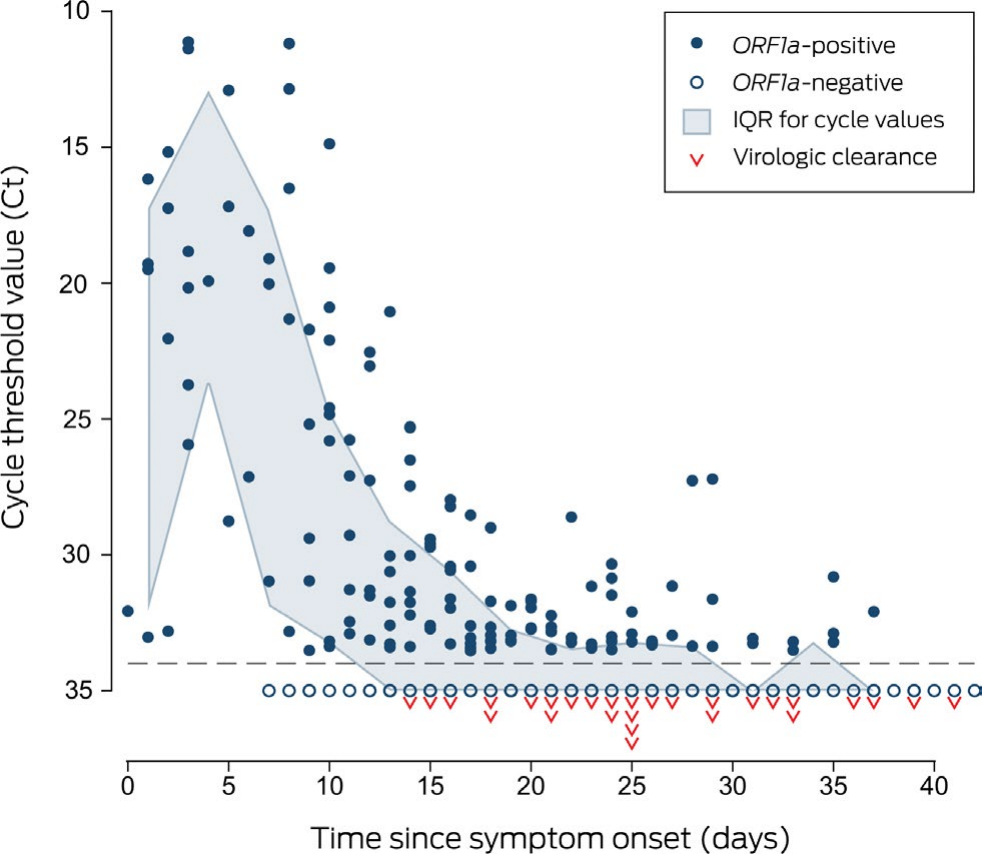

The phylogeny of the 27 available NT viral genomes was consistent with acquisition in locations on all inhabited continents (Box 3). Five genetic clusters were evident (maximum of one single nucleotide polymorphism within each cluster) that were also epidemiologically linked by shared travel or household contact. The SARS‐CoV‐2 genomes from two independent travellers without epidemiologic connections were identical, but matched other publicly available genomes, highlighting the importance of interpreting genomic analyses in their epidemiologic context.

Box 3 **Maximum likelihood phylogenetic tree, depicting SARS‐CoV‐2 genomes from the Northern Territory and elsewhere**

SARS‐CoV‐2 = severe acute respiratory syndrome coronavirus 2.

The phylogenetic tree shows that SARS‐CoV‐2 genomes in the Northern Territory (on the inner side of the outer ring) were drawn from across the range of genomes reported elsewhere (outer ring). NT travel‐related cases with epidemiologic links formed genomic clusters. Two cases without epidemiologic links also comprised a cluster, but the genomes were identical with overseas genomes. The context genomes were obtained from GISAID (https://www.gisaid.org), with region based on location of the submitting laboratory; the Wuhan‐Hu‐1 genome was used as an outgroup, and the scale bar indicates substitutions per site.

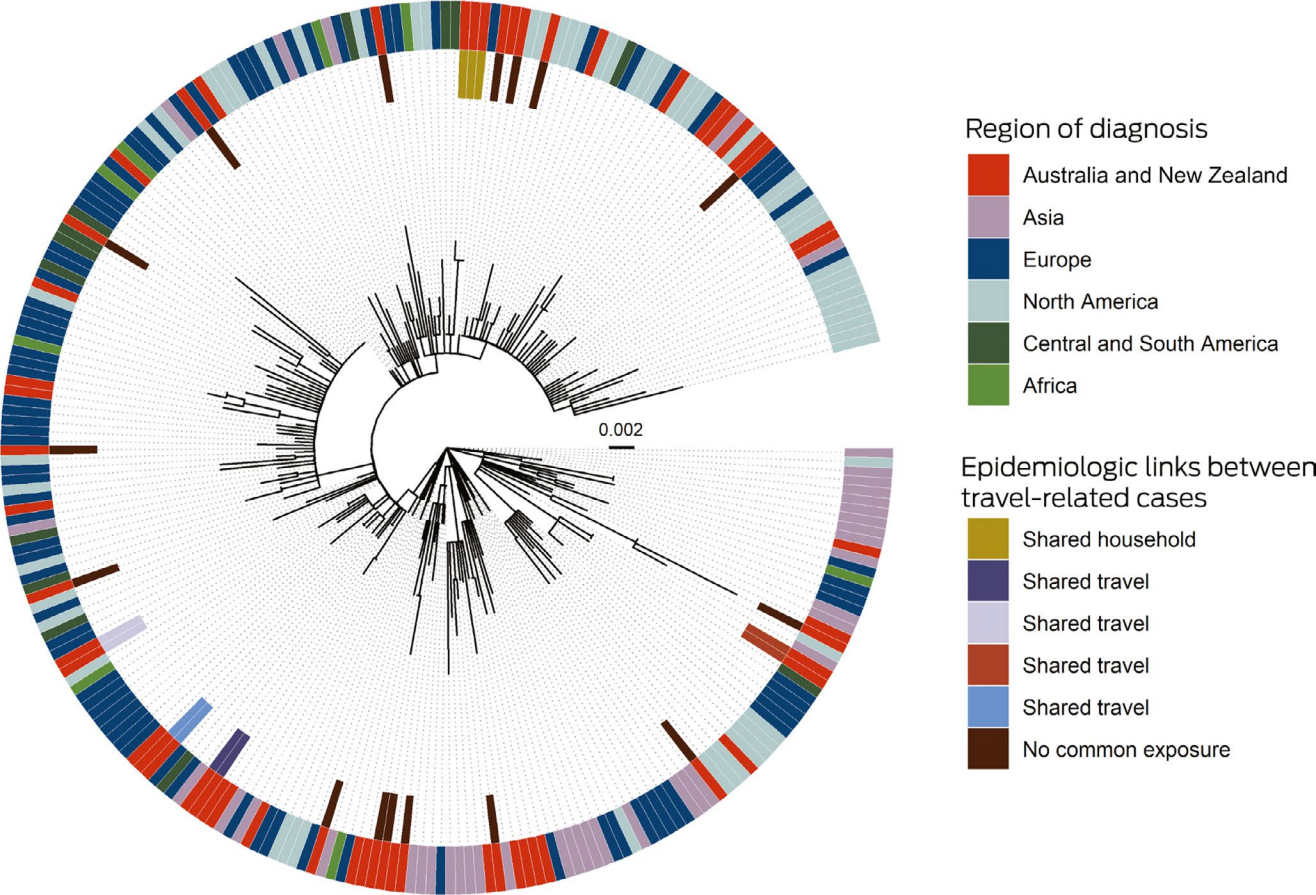

The priority of the strict NT isolation requirements for patients with COVID‐19 was viral containment at a time when data on the duration of viral transmissibility were sparse. More recent evidence suggests that viable SARS‐CoV‐2 is rarely isolated more than 10 days after symptom onset,^1^, ^2^, ^3^ and requirements have consequently been eased, while maintaining supervised isolation with health management during the period of greatest infectivity. The high degree of temporal variability in viral shedding during follow‐up indicates that a single assay is not adequate for excluding infection in patients at epidemiologic risk of COVID‐19.

The NT implemented particularly aggressive public health measures to contain SARS‐CoV‐2 transmission. Epidemiologic and genomic analyses suggest that this response has successfully prevented local community transmission of the virus.

## Competing interests

No relevant disclosures.

## Supporting information

The Northern Territory COVID‐19 Response Group

Methodology and supplementary results
