# Supplementary material for: Successful containment to date of SARS‐CoV‐2 transmission in the Northern Territory
Source: Med J Aust. 2020 Oct 28;214(5):218–9. doi: 10.5694/mja2.50840 (PMC7984294; doi:10.5694/mja2.50840)
Supplement: Supplementary file 1 — The Northern Territory COVID‐19 Response Group [file MJA2-214-218-s001.pdf]

The Medical Journal of Australia

Supporting Information 1

Methodology and supplementary results

This appendix was part of the submitted manuscript and has been peer reviewed.
It is posted as supplied by the authors.

Appendix to: Douglas NM, Meumann EM, Krause VL, Davies J, for the Northern Territory COVID-19 Response Group. Successful containment to date of SARS-CoV-2 transmission in the Northern Territory. *Med J Aust* 2021; doi: 10.5694/mja2.50840.

Public health response and clinical, laboratory and genomic methodology

Clinical and public health processes

The NT Centre for Disease Control (NTCDC) issued its first NT COVID-19 public health alert on 24 January 2020. Twenty-four subsequent alerts[1] have mostly followed the Australian Series of National Guidelines[2] with the exception that testing criteria have been broader and isolation procedures more conservative. All patients with coronavirus disease 2019 (COVID-19) were placed in mandated, supervised isolation until two negative severe acute respiratory syndrome coronavirus 2 (SARS-CoV-2) swabs had been documented at least 24 hours apart. This isolation requirement was formulated prioritising viral containment at a time of limited data on duration of infectiousness. Surveillance testing of all hospitalised patients with respiratory illness and/or unexplained fever was commenced on March 3. Contact tracing and quarantine of close case contacts coordinated by the NTCDC has been rigorous and near complete. Pandemic clinics for assessing and swabbing suspect cases were mobilised rapidly in various locations.

All patients diagnosed with COVID-19 in the Northern Territory were admitted to hospital for initial assessment by an infectious diseases team where possible. Blood and radiological investigations were at the discretion of the admitting doctors but were influenced by emerging evidence on relevant prognostic laboratory markers.[3,4,5]

Once clinically stable, patients were transferred to an external supervised isolation facility. Daily clinical assessment continued to occur at these facilities. By departmental consensus, patients with COVID-19 could be treated with lopinavir-ritonavir 400/100mg once daily for up to 5 days provided patient consent was obtained. The ASCOT trial was not available for enrolment[6] whereas the REMAP-CAP trial[7] was available for patients admitted to the intensive care unit in Darwin.

Laboratory procedures

Diagnosis of SARS-CoV-2 infection was made by detection of SARS-CoV-2 RNA on dry flocked swabs (Copan, Italy) collected from the oropharynx and bilateral deep nasal passages, using one of two multiplex tandem polymerase chain reaction (PCR) assays from AusDiagnostics (Australia). Until mid-March, a coronavirus typing panel was used with primers for the open reading frame 1a (ORF1a) gene target specific for SARS-CoV-2. From mid-March onwards, an updated panel was used which included primers for the ORF1a and open reading frame 8 (ORF8) genes on SARS-CoV-2 as well as targets for influenza and respiratory syncytial virus. The amount of viral RNA present was estimated using the cycle threshold (Ct) value with lower numbers suggesting higher viral loads.

All initial positive assays were verified at the Victorian Infectious Diseases Reference Laboratory with 100% result concordance.

Genomic sequencing

SARS-CoV-2 genomic sequencing was undertaken at the Microbiological Diagnostic Unit and Public Health Laboratory in Melbourne, Australia. RNA extraction was done using either the Qiagen QIAsymphony DSP Virus/Pathogen Kit or the QIAamp 96 Virus QIAcube HT Kit, and tiled amplicons were prepared using either ARTIC version 1 or version 3 primers (https://github.com/artic-network/artic-ncov2019/tree/master/primer_schemes/nCoV-2019) with published protocols (https://www.protocols.io/view/ncov-2019-sequencing-protocol-bbmuik6w). Library preparation was done with NexteraXT kits and sequencing was done using Illumina NextSeq500/550 or iSeq100 platforms with 150bp paired-end reads.

Statistical analysis

Data preparation, analysis and graphing were done using STATA 15.1 and R 3.6.0. Negative SARS-CoV-2 assays were arbitrarily assigned a Ct value of 35 for graphing purposes. The Kaplan-Meier method was used to present the cumulative probability of virologic clearance by time. All analyses were descriptive and therefore, tests of statistical significance were not done.

Bioinformatic analysis

Consensus sequences were generated by aligning reads to the Wuhan-Hu-1 (Genbank MN908947.3) reference genome with minimap2 v2.17,[8] and using the output of samtools4 mpileup v1.10[9] with ivar consensus v2.17.[10] The consensus sequences for the 27 NT SARS-CoV-2 genomes were uploaded to GISAID (https://www.gisaid.org, Supplementary Table 2). The 27 NT SARS-CoV-2 genomes were included in a phylogenetic analysis with 237 publicly available Australian and international genomes from GISAID for comparison (Supplementary Table 3), with the exception of one genome which did not meet GISAID's quality requirements due to a 2-nucleotide gap. Only publicly available genomes with >29,000bp length, <1% Ns and <0.05% unique amino acid mutations were selected. Genomes were aligned to the Wuhan-Hu-1 reference genome using MAFFT v7.464.[11] The 5' and 3' untranslatable regions were trimmed from the alignment. Phylogenetic analysis was undertaken using IQ-TREE v1.6.12[12] with options "-mset HKY,TIM2,GTR -mfreq F -5 mrate G,R -alrt 1000 -bb 1000". Outlying genomes on unexpectedly long branches of the tree were excluded from the final analysis based on visual inspection. The tree was annotated using the ggtree package[13] in R v3.6.0. The lineage of the genomes was determined using Pangolin v1.1.11 (https://github.com/cov-lineages/pangolin).[14]

References

1. Northern Territory Department of Health. COVID-19 alert for clinicians. https://health.nt.gov.au/health-alerts/covid-19-alert-for-clinicians (viewed July 2020).

2. Australian Department of Health. Series of National Guidelines (SoNGs). https://www1.health.gov.au/internet/main/publishing.nsf/Content/cdnasongs.htm (viewed July 2020).

3. Li X, Xu S, Yu M, et al. Risk factors for severity and mortality in adult COVID-19 inpatients in Wuhan. *J Allergy Clin Immunol* 2020; 146: 110-118.

4. Zheng Z, Peng F, Xu B, et al. Risk factors of critical & mortal COVID-19 cases: a systematic literature review and meta-analysis. *J Infect* 2020; 81: e16–e25.

5. Wan S, Xiang Y, Fang W, et al. Clinical features and treatment of COVID-19 patients in northeast Chongqing. *J Med Virol* 2020; 92: 797-806.

6. Denholm JT, Davis J, Paterson D, et al. The Australasian COVID-19 Trial (ASCOT) to assess clinical outcomes in hospitalised patients with SARS-CoV-2 infection (COVID-19) treated with lopinavir/ritonavir and/or hydroxychloroquine compared to standard of care: a structured summary of a study protocol for a randomised controlled trial. *Trials* 2020; 21: 646.

7. Angus DC, Berry S, Lewis RJ, et al. The randomized embedded multifactorial adaptive platform for community-acquired pneumonia (REMAP-CAP) study: rationale and design. *Ann Am Thorac Soc* 2020; 17: 879–891.

8. Li H. Minimap2: pairwise alignment for nucleotide sequences. *Bioinforma Oxf Engl* 2018; 34: 3094–3100.

9. Li H, Handsaker B, Wysoker A, et al. The sequence alignment/map format and SAMtools. *Bioinforma Oxf Engl* 2009; 25: 2078–2079.

10. Grubaugh ND, Gangavarapu K, Quick J, et al. An amplicon-based sequencing framework for accurately measuring intrahost virus diversity using PrimalSeq and iVar. *Genome Biol* 2019; 20: 8.

11. Katoh K, Misawa K, Kuma K, Miyata T. MAFFT: a novel method for rapid multiple sequence alignment based on fast Fourier transform. *Nucleic Acids Res* 2002; 30: 3059–3066.

12. Nguyen L-T, Schmidt HA, von Haeseler A, Minh BQ. IQ-TREE: a fast and effective stochastic algorithm for estimating maximum-likelihood phylogenies. *Mol Biol Evol* 2015; 32: 268–274.

13. Yu G, Lam TT-Y, Zhu H, Guan Y. Two methods for mapping and visualizing associated data on phylogeny using Ggtree. *Mol Biol Evol* 2018; 35: 3041–3043.

14. Rambaut A, Holmes EC, O'Toole Á, et al. A dynamic nomenclature proposal for SARS-CoV-2 lineages to assist genomic epidemiology. *Nat Microbiol* 2020; doi: 10.1038/s41564-020-0770-5 [online ahead of print].

Table. Demographic and clinical characteristics of the Northern Territory COVID-19 series (28 patients)

**Characteristic**	
Age	
Median (IQR)	45.0 (29.3–55.4)
Range	1.5–75
Sex	
Male	12 (43%)
Female	16 (57%)
Indigenous status	
Aboriginal/Torres Strait Islander	0
Non-Indigenous	28 (100%)
Presumed country/region of acquisition	
Australia	3 (12%)
United Kingdom	5 (18%)
USA	4 (16%)
Austria	3 (12%)
Sub-Saharan Africa	3 (12%)
At sea	3 (12%)
Indonesia	1 (4%)
South America	2 (8%)
New Zealand	1 (4%)
Canada	1 (4%)
Philippines	1 (4%)
United Arab Emirates	1 (4%)
Symptoms	
Fever	14 (50%)
Coryza	14 (50%)
Cough	13 (46%)
Sore throat	13 (46%)
Dyspnoea	7 (25%)
Myalgia	5 (18%)
Anosmia	5 (18%)
Headache	4 (14%)
Diarrhoea	3 (11%)
Back pain	3 (11%)
Nausea	3 (11%)
Dysgeusia	2 (7%)
Chest pain	2 (7%)
Laboratory parameters, median (IQR)	
Lymphocyte count (RR, 1.5–4.0 × 10^9/L) (*n* = 25)	1.7 (1.5–2.2)
C-reactive protein (RR, < 5 mg/L) (*n* = 25)	2.2 (0.4–5.0)
Aspartate aminotransferase (RR, 5–41 U/L) (*n* = 14)	27.5 (24.0–42.0)
Ferritin (RR, 20–150 µg/L) (*n* = 24)	157 (97–247)
Lactate dehydrogenase (RR, 120–250 U/L) (*n* = 23)	196 (167–208)
D-dimer (RR, ≤ 0.50 mg/L) (*n* = 22)	0.25 (< 0.19–0.39)
Severity (treatment required)	
Supplemental oxygen	2 (7%)
Invasive ventilation	1 (4%)
Antiviral treatment	
Lopinavir–ritonavir	2 (7%)
None	26 (93%)

IQR = interquartile range; RR = reference range.

Figure. Trajectory of polymerase chain reaction (PCR) cycle threshold (Ct) values for the open reading frame 8 (*ORF8*) target based on results of the AusDiagnostics multiplex tandem PCR assay

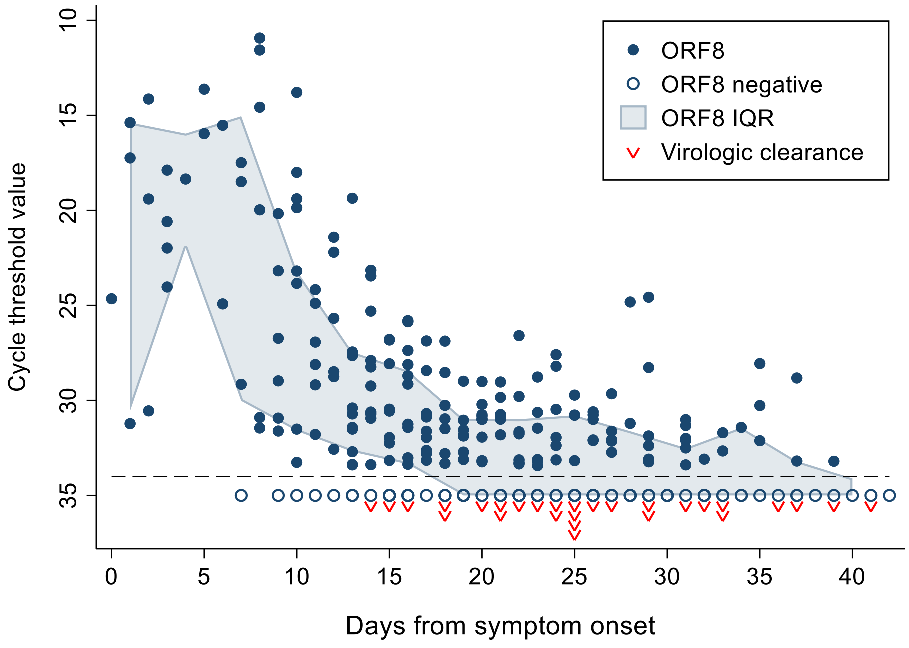

IQR = interquartile range.
