# Supplementary material for: Successful containment to date of SARS‐CoV‐2 transmission in the Northern Territory
Source: Med J Aust. 2020 Oct 28;214(5):218–9. doi: 10.5694/mja2.50840 (PMC7984294; doi:10.5694/mja2.50840)
Supplement: Supplementary file 2 — Methodology and supplementary results [file MJA2-214-218-s002.pdf]

MJA
The Medical Journal of Australia

Supporting Information 2

The Northern Territory COVID-19 Response Group

This appendix was part of the submitted manuscript and has been peer reviewed. It is posted as supplied by the authors.

Appendix to: Douglas NM, Meumann EM, Krause VL, Davies J, for the Northern Territory COVID-19 Response Group. Successful containment to date of SARS-CoV-2 transmission in the Northern Territory. *Med J Aust* 2021; doi: 10.5694/mja2.50840.

Northern Territory COVID-19 Response Group: author details

Nicholas M Douglas[1,2,3]

Ella M Meumann[2,3]

Martin Hansen[3]

Nilanthy Vigneswaran[3]

Melissa Carroll[3]

Abigail Miller[3]

Kevin Freeman[1]

Farshid Dakh[1]

Ouli Xie[4,5]

Shereen Labib[4]

Marcus Clarke[3]

Anna Watson[3]

Aadith Ashok[6]

Richard Budd[7]

Sophie Carey[4]

Sonja Janson[3]

Sarah A Lynar[3,5]

Emma Spencer[3]

Anna P Ralph[2,3]

Patiyan Andersson[8]

Norelle L Sherry[8]

Torsten Seemann[8]

Leon Caly[9]

Benjamin P Howden[8]

Dianne P Stephens[10,11]

Hugh Heggie[11]

Emma Divilly[12]

Heni Hongara[12]

Carol Mackrow[12]

Amy Legg[5,13]

Jennifer Yan[2,14]
Joshua R Francis[2,14]
Lewis Campbell[15]
Didier Palmer[16]
Ric N Price[2,3]
Bart J Currie[2,3]
Belinda Greenwood-Smith[4]
Lloyd Einsiedel[6]
Fabian Chiong[6]
Samuel Goodwin[17]
Nadarajah Kangaharan[18]
Peter Markey[4]
Rob W Baird[1,3]
Catherine S Marshall[3]
Vicki L Krause[4]
Jane Davies[2,3]

1. Territory Pathology, Royal Darwin Hospital, Top End Health Service, Northern Territory Government, Darwin, NT, Australia
2. Global and Tropical Health Division, Menzies School of Health Research, Charles Darwin University, Darwin, NT, Australia
3. Department of Infectious Diseases, Royal Darwin Hospital, Top End Health Service, Northern Territory Government, Darwin, NT, Australia
4. Northern Territory Centre for Disease Control, Public Health Unit, Northern Territory Government, Darwin, NT, Australia
5. Menzies School of Health Research, Charles Darwin University, Darwin, NT, Australia
6. Department of Infectious Diseases, Alice Springs Hospital, Central Australia Health Service, Northern Territory Government, Alice Springs, NT, Australia

7. Department of Medicine, Katherine Hospital, Top End Health Service, Northern Territory Government, Katherine, NT, Australia
8. Microbiological Diagnostic Unit Public Health Laboratory, Department of Microbiology & Immunology, University of Melbourne at the Doherty Institute, Melbourne, VIC, Australia
9. Victorian Infectious Diseases Reference Laboratory, Melbourne, VIC, Australia
10. National Critical Care and Trauma Response Centre, Darwin, NT, Australia
11. Department of Health, Northern Territory Government, Darwin, NT, Australia
12. Department of Nursing, Royal Darwin Hospital, Top End Health Service, Northern Territory Government, Darwin, NT, Australia
13. Department of Pharmacy, Royal Darwin Hospital, Top End Health Service, Northern Territory Government, Darwin, NT, Australia
14. Department of Paediatric Infectious Diseases, Royal Darwin Hospital, Top End Health Service, Northern Territory Government, Darwin, NT, Australia
15. Department of Intensive Care, Royal Darwin Hospital, Top End Health Service, Northern Territory Government, Darwin, NT, Australia
16. Emergency Department, Royal Darwin Hospital, Top End Health Service, Northern Territory Government, Darwin, NT, Australia
17. Alice Springs Hospital, Central Australia Health Service, Northern Territory Government, Alice Springs, NT, Australia
18. Department of Medicine, Royal Darwin Hospital, Top End Health Service, Northern Territory Government, Darwin, NT, Australia
